# Research progress on the causes and countermeasures of postoperative swelling in gluteal muscle contracture

**DOI:** 10.3389/fmed.2025.1592911

**Published:** 2025-09-05

**Authors:** Yuxiang Ren, Li Yang, Yingyi Fan, Bixia Chen, Yanrong Tan, Sha Hu, Jiuqun Li, Cong Yang

**Affiliations:** ^1^Shenzhen Hospital, Peking University, Shenzhen, China; ^2^College of Medicine, Shantou University, Shantou, China

**Keywords:** gluteus contracture, swelling, hematoma, nursing, summary

## Abstract

Surgical site swelling represents a prevalent complication following gluteal contracture release that adversely impacts functional recovery; while current literature predominantly focuses on rehabilitation outcomes, insufficient attention has been paid to swelling etiology. This review synthesizes causative evidence across three determinant categories: (1) preoperative factors—individual variations in coagulation profiles and symptom severity; (2) intraoperative variables—technical disparities in hemostasis, surgical trauma magnitude, and irrigation parameters; (3) postoperative divergences—inconsistent compression bandaging, exercise initiation timing, education quality, and compliance. Critical preventive strategies include implementing comprehensive preoperative education, standardizing surgical protocols, instituting graded rehabilitation, and establishing early detection-intervention frameworks, which collectively reduce swelling incidence and severity. These evidence-based approaches offer optimized clinical pathways while informing future research.

## 1 Introduction

Gluteal muscle contracture (Gluteal muscle contracture, GMC) is a distinct clinical condition marked by the abnormal shortening and tightening of the gluteal muscles, tensor fascia lata (TFL), iliotibial band (ITB), and associated fascia. This condition can lead to restricted movement and discomfort, particularly affecting the external rotation of the hip joint (1). While GMC is a global issue, it exhibits a higher prevalence in China, primarily affecting infants and adolescents (2). Most patients have a history of frequent buttock injections during their early years, with an estimated childhood incidence rate ranging from 1 to 2.5% (3, 4). Currently, both domestically and internationally, the standard treatment for gluteal muscle contracture is the gluteal muscle contracture release surgery. This surgical approach demonstrates distinct advantages over traditional methods and has gained widespread clinical adoption (5). Studies indicate that arthroscopic hip muscle contracture release has become the gold standard globally for treating this condition (6, 7).

Postoperative symptoms such as hematoma and swelling following gluteal muscle contracture surgery can lead to adverse outcomes, including pain, infection, and necrosis, thereby prolonging recovery time and increasing treatment costs (8). Although most literature suggests that postoperative hematoma is typically transient, targeted interventions are still necessary to mitigate potential complications. Studies indicate that postoperative hematomas can lead to severe complications, causing not only pain but also potential nerve damage, which in turn prolongs recovery time and increases treatment complexity and costs (9, 10). The assessment of postoperative hematomas is essential. Studies have suggested two classification methods for surgical wound hematomas after total hip arthroplasty, with the objective classification based on measuring hematoma area demonstrating greater reliability (11). This approach offers a useful reference for evaluating muscle contracture hematomas following hip replacement. Precise assessment enables clinicians to develop more targeted treatment strategies, reducing adverse effects such as swelling and lowering the risk of complications like infection and necrosis. Ultimately, this method shortens recovery time and helps manage treatment costs.

In conclusion, the risks of hematoma and swelling following gluteal muscle contracture surgery should not be underestimated. Although most postoperative hematomas are temporary, clinicians must adopt targeted measures to alleviate patient discomfort, reduce complication rates, accelerate recovery, and lower treatment costs. These measures include optimizing surgical techniques to prevent complications, conducting precise hematoma assessments, and implementing timely interventions. This study reviews the literature to analyze the causes of swelling following gluteal muscle contracture surgery. Targeted solutions are proposed based on these causes, aiming to provide scientific evidence for clinical treatment, optimize therapeutic strategies, effectively reduce postoperative swelling, promote recovery, and enhance the patient’s quality of life.

## 2 Basic theories of postoperative swelling in gluteal muscle contracture

### 2.1 Pathophysiology of gluteal muscle contracture

Gluteal muscle contracture (GMC) is a chronic fibrotic disease of the gluteal muscles with multiple etiologies. Research has shown that in GMC tissues, compared to normal muscle tissues, there are significant changes at the molecular and cellular levels. For instance, the protein and mRNA expression of Emilin 1 are decreased, while miR - 491 - 5p shows an aberrant elevation, and these two are negatively correlated (12). The direct binding of miR - 491 - 5p to Emilin 1 mRNA, as confirmed by luciferase reporter gene assay, leads to the inhibition of Emilin 1 expression. This, in turn, promotes the proliferation and fibrosis of contraction band (CB) fibroblasts via the TGF - β1/Smad3 signal axis.

FKBP prolyl isomerase 10 (FKBP10) also plays a key role in GMC progression. In the gluteal muscle of GMC patients and rats, FKBP10 expression is up - regulated, accompanied by obvious tissue damage and fibrosis. Elevated levels of TGFβ1, *α* - SMA, collagen I, collagen III, vimentin, fibronectin, p62, and LC3, along with decreased levels of MMP9 and LC3II/I, Beclin 1, p62, and ATG7, indicate weakened autophagy (13). The interaction between FKBP10 and HSP47 further inactivates the HSP47/SMAD3 signaling pathway, which is crucial in the development of GMC.

### 2.2 Mechanisms of postoperative swelling

The mechanisms of postoperative swelling in gluteal muscle contracture are complex and multifactorial. Inflammation is a common contributing factor. After surgery, the body’s immune response is activated, leading to the release of pro - inflammatory cytokines such as interleukin 1β (IL - 1β), interleukin 18 (IL - 18), and tumor necrosis factor *α* (TNF - α) (14). These cytokines can increase vascular permeability, allowing fluid to leak into the interstitial space and cause swelling.

Another aspect is related to the disruption of blood vessels during surgery. Inosculation of blood vessels is important for the early perfusion and vitality of tissues. However, if the normal process of vascular inosculation is disrupted, it may lead to ischemia in the surgical area, followed by reperfusion injury, which can also contribute to swelling (15). Additionally, factors such as fluid overload, exogenous opioids, neurohormonal dysfunction, and gastrointestinal stretch and inflammation, which are key mechanisms in the pathophysiology of postoperative ileus in general surgeries, may also play a role in the postoperative swelling of gluteal muscle contracture surgeries (16).

## 3 Analysis of the causes of postoperative swelling in the surgical area

### 3.1 Preoperative individual differences

Preoperative coagulation function and the severity of patient symptoms directly influence surgical outcomes and postoperative swelling. Patients with poor coagulation function require more extensive preoperative preparation. Intraoperative trauma can damage the vascular endothelial tissue, impairing venous blood flow and resulting in reduced postoperative activity compared to preoperative levels. This can lead to slow blood circulation, making patients more susceptible to bruising and severe swelling (17). Shrestha et al. classified gluteal contracture into four types based on the hip inversion angle during 90° hip flexion and 90° knee flexion: type I (−5° to −20°), type II (−20° to −40°), type III (−40° to −60°), and type IV (> − 60°). As the degree of hip inversion increases, clinical symptoms worsen, making surgery more challenging and outcomes less favorable (17, 18). This classification method is valuable for predicting preoperative surgical outcomes.

### 3.2 Critical intraoperative procedures

#### 3.2.1 Degree of subcutaneous adipose tissue removal

During arthroscopic release of gluteal contracture, the creation of an operative space between the subcutaneous adipose layer and the fibrous contracture bands requires precise dissection. Inadequate debridement of adipose tissue may obscure the arthroscopic view, increasing the risk of iatrogenic injury to neurovascular structures (19). Conversely, excessive adipose destruction can damage the vascularized gluteal fascia and underlying muscle fibers, triggering inflammatory exudation that predisposes to postoperative hematoma or seroma formation (20). Furthermore, when the gluteal contracture band is located in deep anatomical layers or when uncontrolled intraoperative hemorrhage compromises arthroscopic visualization, conversion to open surgery may be necessitated for safety preservation (21). However, open approaches require extensive surgical dissection, resulting in significantly higher intraoperative blood loss (mean: 185 ± 32 mL vs. 45 ± 15 mL in arthroscopy) (22). The limited tactile feedback and optical resolution in distinguishing neurovascular structures under direct visualization increase susceptibility to iatrogenic injury, particularly to the superior gluteal neurovascular bundle traversing the intermuscular plane (23).

#### 3.2.2 Intraoperative irrigation

Management Continuous high-volume irrigation is essential for maintaining a clear surgical field during arthroscopic gluteal contracture release. However, excessive irrigation fluid absorption may induce localized tissue swelling and postoperative gluteal pain (24). The primary complication stems from fluid extravasation into interstitial spaces, where an elevated extracellular osmotic gradient (>290 mOsm/L) relative to intracellular levels triggers fluid influx into cells, causing intraoperative cellular edema (25). As detailed below, elevated perfusion pressure (>40 mmHg) disrupts fluid homeostasis through a triple cascade: (1) It directly increases capillary hydrostatic pressure (Pc) and dilutes plasma colloid osmotic pressure (πp), overriding Starling forces to drive fluid extravasation; (2) Hyperosmotic irrigants (>290 mOsm/L) induce transmembrane osmotic shock, triggering cellular edema via ENaC-mediated water influx and impairing post-reversal membrane repair; (3) Mechanotransduction (Piezo1/Ca^2+^-VEGF axis) and inflammatory mediators further degrade endothelial barrier integrity, perpetuating protein-rich fluid accumulation. These mechanisms collectively elucidate how pressure disrupts osmotic equilibrium and exacerbates tissue swelling (26). Postoperatively, reversal of this osmotic gradient facilitates fluid extravasation from damaged cells into the periarticular tissue space. Given the higher density of periarticular connective tissue compared to joint cavities, elevated irrigation pressures (>40 mmHg) are often required for adequate visualization during microscopic dissection (27). Nevertheless, such pressures directly correlate with interstitial fluid accumulation, exacerbating tissue edema through mechanotransduction-mediated vascular permeability enhancement.

#### 3.2.3 Intraoperative hemostasis

Extensive release of fibrotic bands in gluteal contracture necessitates significant dissection through normal muscular tissue, resulting in postoperative gluteal weakness and elevated risk of neurovascular injury within the gluteal compartment (25). The retraction of transected contracture tissues creates inevitable dead spaces, while the highly vascularized gluteal fascia predisposes to intraoperative damage of gluteus maximus fibers. Consequently, persistent wound bleeding occurs due to incomplete intraoperative hemostasis (28). Furthermore, the combination of a deep surgical field with limited access, residual irrigation fluid accumulation, traumatic exudation, and ineffective postoperative drainage (e.g., tube obstruction) promotes hematoma formation. If such hematomas organize into fibrin-rich clots triggering scar contracture, surgical re-intervention may be required (29).

### 3.3 Timing of postoperative functional exercise

Premature postoperative mobilization may exacerbate pain, swelling, and inflammatory exudates at the surgical site by amplifying tissue inflammatory responses and increasing hemorrhage risk. A bidirectional interaction exists between pain and edema, mediated by the body’s stress response: pain stimulates neuroendocrine release of catecholamines and cortisol, which enhance vascular permeability and aggravate tissue edema (30). Conversely, edema compromises microcirculation and induces mechanical compression of neural structures, thereby intensifying pain perception and impeding functional recovery (4).

### 3.4 Patients’ knowledge and beliefs

Insufficient understanding of postoperative recovery principles combined with outcome overexpectation frequently leads to non-adherence to graded rehabilitation protocols. Premature initiation of high-intensity exercises potentiates risks of periarticular soft tissue injury and hematoma formation (31). Biomechanical analysis indicates that elevated suture tension at the distal surgical site—when compounded by aggressive internal rotation maneuvers or uncontrolled hip flexion during the early healing phase—may induce wound deformation. This mechanical stress disrupts microvascular integrity, resulting in inflammatory exudation, expanding hematomas, and delayed tissue healing.

## 4 Postoperative swelling countermeasures in the surgical area

### 4.1 Preoperative physical examination to assess the surgical area

Gluteal contracture release is predominantly performed using arthroscopic techniques, which are characterized by minimal muscle tissue dissection, reduced trauma, and limited exposure (32). Consequently, a thorough preoperative physical examination is essential to assess the extent of the contracture. The surgical area can be divided into a grid pattern based on the anatomical surface projection of the hip, with key anatomical structures marked for surgical guidance (33). This approach helps the surgeon to better understand the local neurovascular anatomy, allowing for the design of a personalized surgical plan that significantly reduces the risk of nerve and blood vessel injury.

### 4.2 Intraoperative avoidance of inadvertent injury to normal muscle fibers and reduction of bleeding

To ensure successful surgery, it’s crucial to minimize bleeding and maintain a clear surgical field. We carefully select the surgical approach, site, and access, avoiding damage to normal muscle fibers, the sciatic nerve, and supragluteal vessels and nerves. The procedure progresses from superficial to deep, cutting contracture bands and bundles obliquely and precisely under a microscope (34). We repeatedly check signs like the “cross-legged sign” and hip popping for complete release. Using 1 mg of epinephrine per 3 L of perfusion solution for continuous flushing helps reduce bleeding and drainage, keeping the field clear and preventing accidental nerve and vessel injury (35).

In gluteal muscle contracture surgery, the blunt puncture head creates a small cavity, requiring the surgeon to be well-versed in anatomy, skilled in arthroscopic techniques, and adept at three-dimensional spatial thinking. The area around the greater trochanter, where the gluteus maximus, iliotibial fascia, and tensor fasciae latae attach, is often targeted for surgical release. Some studies suggest that a modified arthroscopic approach using an “L” or “C” shaped release path around the greater trochanter can minimize subcutaneous separation, reduce neurovascular injury, expand the release area, and speed up recovery (36). This technique involves a minimally invasive excision of the contracture zone with selective release around the greater trochanter, showing good surgical outcomes.

### 4.3 Intraoperative reduction or avoidance of extravasation of irrigation fluid

Accumulation of irrigation fluid in gluteal tissues contributes to postoperative swelling and pain; strict control of irrigation volume and pressure minimizes fluid extravasation into interstitial spaces, thereby reducing hematoma formation. Maintaining appropriate irrigation pressure is critical since insufficient pressure impairs hemostasis, while excessive pressure promotes tissue edema. To sustain consistent cavity pressure, surgical nurses must continuously monitor fluid flow rates and replenish supplies promptly—supported by evidence from shoulder arthroscopy where perfusion pressures reach 50 mmHg (peaking transiently at 120 mmHg) yet normalize within 4–30 min without inducing myofascial compartment syndrome or compromising muscle perfusion (37). Optimal management therefore combines controlled irrigation flow/pressure with postoperative drainage to mitigate swelling from fluid leakage; nevertheless, evidence defining perfusion pressure parameters for gluteal surgery remains limited, and no standardized pressure range exists, warranting further investigation.

### 4.4 Effective hemostasis and adequate drainage during the perioperative period

Inadequate hemostasis or drainage exacerbates postoperative swelling. Effective physical hemostasis includes direct compression and pressure bandaging. Adjunctive cold therapy reduces surgical site bleeding, while layered dressings and positional changes (supine/lateral rotation) prevent hematoma formation (38). Improved compression techniques—such as external wound placement of sandbags secured with abdominal binders—enhance hemostatic efficacy compared to standard sandbag compression.

Compression garments (e.g., elastic pants) provide targeted pressure with improved comfort and mobility, concurrently mitigating rehabilitation pain and preserving patient dignity. Pharmacological approaches, notably topical tranexamic acid applied perioperatively to soft tissues, significantly reduce bleeding, exudate, and swelling, particularly in early postoperative stages (39). Effective drainage mitigates postoperative hematoma risk by evacuating accumulated blood from deep cavities formed by retracted transected tissues during gluteal contracture release—a condition characterized by narrow openings that impair natural drainage. Key interventions include suturing the deep fascial superficial layer to reduce tension, placing drainage tubes into cavity depths to prevent infection and adhesions, and applying negative pressure to maintain tissue apposition for internal hemostasis without increasing blood loss (5).

Synergistic hemostatic approaches beyond conventional methods significantly mitigate hematoma risk. Clinical evidence confirms that adrenaline-infused pumps (0.1% HCl epinephrine) at surgical sites stabilize perioperative circulation and prevent hematoma formation (40). Given that occult blood loss drives postoperative complications—including hematomas, tissue swelling, and pain—combined interventions yield multiplicative benefits: plasma knife coagulation followed by topical tranexamic acid (TXA) in the operative cavity, drainage tube clamping for 3 h post-TXA application, protocolized systemic TXA dosing (20 mg/kg preoperatively; 10 mg/kg at 3- and 6-h intervals), intraoperative TXA-soaked gauze for 10 min before closure, and 24-h compression bandaging collectively reduce blood loss and surgical site morbidity (39).

Therefore, meticulous hemostasis and drainage management are imperative. Early intraoperative pressure bandaging should be applied, with vigilant maintenance of drainage tube patency and surgical site cleanliness. Clinicians must routinely assess and document drainage characteristics (color, viscosity, volume), while instructing patients on position changes to prevent tube dislodgement or compression-induced occlusion.

### 4.5 Reduce or avoid early high-intensity exercise after surgery

Early postoperative mobilization prevents joint adhesions, however, premature high-intensity exercise risks surgical site disruption, exacerbating inflammation, hemorrhage, pain, and swelling. Consequently, standardized progressive rehabilitation protocols are essential to prevent contracture recurrence and promote functional hip recovery (41). Patients should initiate graded exercises with incremental intensity increases during healing to avoid iatrogenic injury—evidenced by clinical reports of uncontrolled activity complications: three hematomas within 10 days postoperatively resulted from abrupt hip flexion/extension with knee fixation, requiring emergent ligation of injured circumflex femoral artery branches; another hematoma confirmed by ultrasonography followed 100 consecutive deep squats on postoperative day 3. Although resolved through intervention, these incidents significantly delayed recovery, underscoring the critical balance between early mobility and controlled progression (42).

Consequently, clinical staff should prioritize patient education on postoperative rehabilitation principles, emphasizing the critical importance of progressive overload training—advancing from low-intensity passive to high-intensity active exercises while maintaining respiratory control (41). This approach establishes active-dominant training supplemented by passive modalities. Real-time correction of improper techniques during supervised sessions prevents bleeding, swelling, and soft tissue injuries caused by erroneous repetitions. Structured education enhances patient self-management capacity, reducing complication risks through protocol adherence.

### 4.6 Postoperative administration of a combination of drugs and physical therapy

Pain and swelling demonstrate bidirectional exacerbation, with severe pain inducing hypothalamic–pituitary–adrenal (HPA) axis dysregulation that impairs appetite, sleep quality, limb mobilization, and surgical site healing (43). To address exercise-limiting pain and anticipatory anxiety, postoperative NSAIDs are routinely administered. Concurrently, clinicians must educate patients on critical rehabilitation principles: (1) premature repetitive exercise risks wound disruption, whereas exercise avoidance compromises functional recovery; (2) structured cognitive restructuring mitigates kinesiophobia. Implementation requires integrating attention-diversion techniques (e.g., controlled breathing > media engagement) with antalgic positioning to enhance comfort—collectively optimizing rehabilitation adherence while reducing iatrogenic injury risks (44).

Adjunctive modalities further mitigate pain and swelling. Physiotherapy interventions—including ultrashort wave, therapeutic ultrasound, or hyperbaric oxygen applied to peri-incisional tissues starting postoperative day 2—enhance microcirculation, reduce inflammation, and accelerate hematoma resorption. Complementary traditional Chinese medicine (TCM) approaches, such as *Crocus sativus* L. infusion and Shujin Huoxue Lotion, demonstrate pharmacodynamic properties that suppress inflammatory exudation, promote hematoma decompression, and alleviate exercise-induced discomfort during early rehabilitation (3, 45).

## 5 Conclusion

Both open surgery and endoscope-assisted techniques improve functional satisfaction in gluteal muscle contracture patients. However, arthroscopic release demonstrates superior outcomes: fewer complications, enhanced cosmesis, smaller incisions, and shorter hospitalization. Minimally invasive arthroscopy constitutes the gold standard for gluteal contracture management, with postoperative complications (e.g., surgical site swelling, subcutaneous hematoma) primarily driven by preoperative patient factors, intraoperative technical variances, and postoperative rehabilitation deviations. To optimize outcomes, surgeons must master procedural anatomy, maintain hemorrhage control and irrigation pressure, and implement hemostasis-drainage synergies—while standardized perioperative care bundles encompassing structured preoperative education, intraoperative positioning precision, dynamic pressure monitoring, postoperative compression dressings, and multimodal analgesia collectively reduce complications and hospitalization duration. Technological advancements now position arthroscopy as routine practice, yet establishing systematic surgical-nursing protocols coupled with patient-centric self-management education remains critical for safety and accelerated rehabilitation. Due to the scant literature on intraoperative gluteal perfusion pressure, our analysis could not explore this parameter in depth. Further investigations should prioritize randomized trials comparing different perfusion pressures during surgery, along with longitudinal studies tracking long-term swelling management outcomes.
