# Correction: Research progress on the causes and countermeasures of postoperative swelling in gluteal muscle contracture

**DOI:** 10.3389/fmed.2025.1716270

**Published:** 2025-10-17

**Authors:** Yuxiang Ren, Li Yang, Yingyi Fan, Bixia Chen, Yanrong Tan, Sha Hu, Jiuqun Li, Cong Yang

**Affiliations:** ^1^Shenzhen Hospital, Peking University, Shenzhen, China; ^2^College of Medicine, Shantou University, Shantou, China

**Keywords:** gluteus contracture, swelling, hematoma, nursing, summary

In the published article, there was a mistake in the **Funding** statement. The funding statement for the 2021 Research project of Peking University Shenzhen Hospital was displayed as “LCYJ2020003”. The correct statement is “Peking University Shenzhen Hospital Research Project (LCYJ2020003).”

In the published article, there was a mistake in the Funding statement. The funding statement for the Shenzhen Basic Research special Project was displayed as “202205303003082”. The correct statement is “Shenzhen Science and Technology Program (JCYJ20220530160212028).”

The funder [Shenzhen Health Economics Project], [202532] to [Yuxiang Ren] was erroneously omitted.

The original article has been updated.

